# Editorial: Shared decision-making in neurology

**DOI:** 10.3389/fneur.2023.1222433

**Published:** 2023-06-06

**Authors:** Christoph Heesen, Alessandra Solari

**Affiliations:** ^1^Institute of Neuroimmunology and Multiple Sclerosis, University Clinic Hamburg-Eppendorf, Hamburg, Germany; ^2^Department of Neurology, University Clinic Hamburg-Eppendorf, Hamburg, Germany; ^3^Department of Research and Clinical Development, Unit of Neuroepidemiology, Fondazione IRCCS Istituto Neurologico Carlo Besta, Milan, Italy

**Keywords:** multiple sclerosis, shared decision making, risk communication, empowerment, patient information

It has been a quarter of a century since Charles' seminal paper on shared-decision making (SDM) in medicine (1). Based on the bioethical principle of patient autonomy which had been to some extent neglected for many years and reemphasized by the world physician association in 2017 (2), SDM is a model in which informed decisions are collaboratively made by physicians and patients based on the best available evidence and the patient's values and preferences (1, 3). Several studies have shown the benefits of SDM, which is considered to be a key component of high-quality healthcare (4). Empirical measures of SDM are associated with affective-cognitive outcomes (e.g., increased patient knowledge and reduced decisional conflict), while evidence is uncertain for behavioral outcomes (e.g., treatment adherence and exercise) and health outcomes (e.g., symptoms, functioning, and physiological outcomes) (5). SDM is especially important in cases of preference-sensitive decisions that typically occur in chronic medical conditions characterized by variable prognoses and the limited effectiveness of disease-modifying treatments. This is the case for most neurological disorders where such decisions are made from diagnostic workup to the end-of-life phase. Starting from genetic testing and pain management (especially headache), epilepsy, multiple sclerosis (MS), and Parkinson's disease represent conditions where many preference-sensitive decisions are made. Stroke and other acute neurological conditions, dementias, and neuropalliative care give substantial challenges but also opportunities to involve patients and their significant others.

Within this Research Topic of Frontiers in Neurology on SDM, it is worth mentioning the Share to Care (S2C) program of the University Hospital Schleswig-Holstein (Kiel, Germany) involving 17 clinics (Stolz-Klingenberg et al.). S2C is a comprehensive program consisting of four intervention modules addressing healthcare professionals (physicians and nurses) and patients, and newly-developed digital evidence-based decision aids. The SDM level before and 6–18 months after S2C implementation within the Kiel Neuromedical Center was assessed in consecutively selected patients, who reported a significant increase in perceived involvement in decision-making. Five key indications were selected: first immunotherapy for multiple sclerosis (MS) vs. “watch and wait”; deep brain stimulation vs. L-Dopa pump for Parkinson's disease; ultrasound vs. deep brain stimulation for severe tremor; selection of antiepileptic treatment; and selection of treatment for neuropathic pain. Importantly, an additional health insurance-based reimbursement currently secures the S2C program maintenance.

MS is a paradigmatic neurological disease where SDM has been studied for now nearly 20 years. Most of the resulting publications are qualitative studies, surveys, and position papers, while very few assess the impact of evidence-based interventions. The current Research Topic reports on a narrative review of SDM in MS (Ubbink et al.), as well as two pilot trials.

Communication about imaging results is a largely neglected research field in medical education in general. Freund et al. report on the development and pilot testing of a web-based magnetic resonance imaging (MRI) education program for people with MS. The program was well received by people with MS, who showed higher MRI risk knowledge and perceived competence compared to non-contemporaneous controls. Finally, the paper by Wenzel et al. describes the development of “Abouts”, a web-based relapse management intervention based on a previous face-to-face program and investigated in the “Power@MS2” trial (6). Corticosteroids are the first-choice treatment for MS relapses; however, there is still considerable variability in the type, route, and schedule of administration (7). Intravenous remains the most used route of administration, despite oral administration showing similar efficacy in reducing impairment and MRI-enhancing lesions. Oral treatment can be preferred to avoid hospital admissions, which was particularly helpful during the pandemic (7, 8). “Abouts” was well accepted by people with MS who also informed the refinement of the tool.

Being aware that complex evidence-based medical decisions need personalized approaches and sufficient time, nurse-delivered coaching programs for patients in preparation for a medical encounter represent a way to deal with the decreased availability of doctors and the increased patient load and treatment options (9). A pilot study has shown a high appreciation of this approach by persons with MS, while structural and cultural attitudes were barriers to its implementation (10).

Besides MS, recently published SDM papers focus on stroke (11), early dementia (12), and rare neurological disorders (13). Finally, improvement in goals-of-care conversations and advance care planning implementation is part of SDM and of utmost importance in neurology (14, 15).

Taken together, Neurology is a paradigmatic field for SDM. Results of disease overarching education on SDM as the S2C project is encouraging and the scientific landscape is open for young researchers to move the field forward. We are on the way.

## Author contributions

CH drafted the manuscript, which AS than substantially streamlined. All authors contributed to the article and approved the submitted version.
